# Stress perfusion CMR reliably risk stratifies patients with prior exercise treadmill testing

**DOI:** 10.1186/1532-429X-13-S1-P119

**Published:** 2011-02-02

**Authors:** Angela G Bertaso, James D Richardson, Adam J Nelson, Dennis Wong1, Hussam Tayeb, Benjamin K Dundon, Payman Molaee, George Awwad, Kerry Williams, Matthew I Worthley, Karen S Teo, Stephen G Worthley

**Affiliations:** 1 Cardiovascular Research Centre, Royal Adelaide Hospital & Department of Medicine, University of Adelaide, Adelaide, Australia; 2 Cardiovascular Research Centre, Royal Adelaide Hospital, Adelaide, Australia

## Objective

To determine the clinical utility of stress perfusion cardiac magnetic resonance (CMR) in patients with prior exercise treadmill test (ETT).

## Background

ETT is an inexpensive and easily accessible non-invasive test. However it affords only modest sensitivity and specificity for ischaemic heart disease (IHD). Accordingly ETT can often provide results that are either equivocal or which run contrary to clinical suspicion. Therefore, clinicians frequently request an additional non-invasive test to clarify matters. Adenosine stress perfusion CMR has been shown to have a high sensitivity and specificity for the detection of IHD. We sought to evaluate a cohort of patients who had a recent ETT followed by stress perfusion CMR to examine the clinical utility of CMR in risk stratifying these patients.

## Methods

Retrospective analysis of patients referred for adenosine stress perfusion CMR in whom an ETT had been performed in the previous three months. Perfusion imaging was obtained at stress (adenosine 140 µg/kg/min) and rest on a 1.5T scanner. Late enhancement was assessed with dual pass gadolinium (0.2mmol/kg total dose). Patient records, hospital databases and national death registries were reviewed. Major adverse cardiac events (MACE) comprising death, myocardial infarction, revascularisation or ischaemic hospitalisation were evaluated.

## Results

Seventy patients (50% male, age 58.4 years ±11.2; mean ±SD) were identified. Twelve (17%) had a history of previous MI or revascularisation. Seventeen had an objectively positive ETT, of which 7 (41%) had a positive stress CMR. Thirty one had an equivocal ETT, of which 8 (26%) had a positive stress CMR. Twenty two had a negative ETT, of which 3 (14%) had a positive CMR. All positive CMRs had stenoses ≥50% at angiography, except for two patients (11%), both from the equivocal ETT group. A negative CMR, irrespective of ETT status, resulted in a 0% MACE rate at median 23 months (IQR 20-27) follow up. No positive CMRs were seen in negative or equivocal ETTs where ≥ 10 metabolic equivalents (METS) were attained.

## Conclusions

Adenosine stress perfusion CMR can reliably risk stratify patients independent of previous ETT result. A negative CMR is associated with an excellent prognosis at a median of 23 months follow up.

